# Mechanisms of increased *Trichodesmium* fitness under iron and phosphorus co-limitation in the present and future ocean

**DOI:** 10.1038/ncomms12081

**Published:** 2016-06-27

**Authors:** Nathan G. Walworth, Fei-Xue Fu, Eric A. Webb, Mak A. Saito, Dawn Moran, Matthew R. Mcllvin, Michael D. Lee, David A. Hutchins

**Affiliations:** 1 Marine and Environmental Biology, Department of Biological Sciences, University of Southern California, 3616 Trousdale Parkway, Los Angeles, California 90089, USA; 2 Marine Chemistry and Geochemistry Department, Woods Hole Oceanographic Institution, Woods Hole, Massachusetts 02543, USA

## Abstract

Nitrogen fixation by cyanobacteria supplies critical bioavailable nitrogen to marine ecosystems worldwide; however, field and lab data have demonstrated it to be limited by iron, phosphorus and/or CO_2_. To address unknown future interactions among these factors, we grew the nitrogen-fixing cyanobacterium *Trichodesmium* for 1 year under Fe/P co-limitation following 7 years of both low and high CO_2_ selection. Fe/P co-limited cell lines demonstrated a complex cellular response including increased growth rates, broad proteome restructuring and cell size reductions relative to steady-state growth limited by either Fe or P alone. Fe/P co-limitation increased abundance of a protein containing a conserved domain previously implicated in cell size regulation, suggesting a similar role in *Trichodesmium.* Increased CO_2_ further induced nutrient-limited proteome shifts in widespread core metabolisms. Our results thus suggest that N_2_-fixing microbes may be significantly impacted by interactions between elevated CO_2_ and nutrient limitation, with broad implications for global biogeochemical cycles in the future ocean.

## Abstract (web summary)

Cyanobacterial nitrogen fixation supplies bioavailable nitrogen to marine ecosystems, but the mechanisms governing iron and phosphorus co-limitation in elevated CO_2_ remain unknown. Here, the authors show a complex cellular response to co-limitation characterized by changes in growth, cell size, and the proteome.

## Graphical abstract

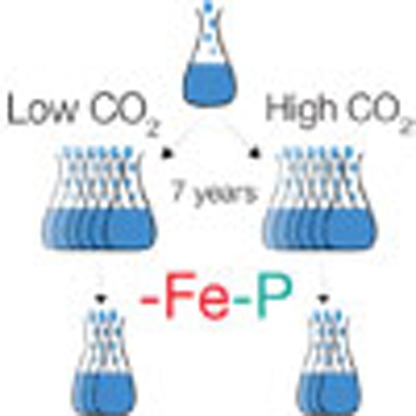

Biological atmospheric nitrogen (N_2_) fixation by cyanobacteria including filamentous *Trichodesmium* spp. is a globally significant biogeochemical process, as it contributes a major fraction of the new N supporting food webs in ocean basin-scale ecosystems^1^,^2^,^3^,^4^. Although prevailing N limitation of the central gyre ecosystems provides an important ecological niche for diazotrophic cyanobacteria, field data have demonstrated iron (Fe) or phosphorus (P) limitation of N_2_ fixation in both the Atlantic and Pacific Oceans^1^,^5^,^6^,^7^,^8^,^9^.

Traditionally, biomass limitation by the single nutrient in shortest supply (that is, Liebig limitation^10^) has been invoked as the controlling mechanism for marine primary production and carbon sequestration^11^,^12^ (see Supplementary Note 1 for more discussion). Depletion of this primary limiting nutrient can then lead to a secondary limitation by the next most limiting nutrient.

More recently, several studies have demonstrated nutrient co-limitation, whereby two nutrients can limit growth simultaneously rather than sequentially^1^,^13^,^14^,^15^,^16^. These observations suggest that marine microbes persistently experience periods of selective pressure under widespread nutrient co-limitation^17^, which may have favoured the evolution of specific metabolic responses to co-limiting conditions. For instance, diazotrophic cyanobacteria simultaneously limited by Fe and P (Fe/P co-limitation) grow and fix N_2_ faster than when limited by either nutrient alone, suggesting that they may possess adaptations specific to co-limited oligotrophic environments^18^.

Despite the apparent importance of co-limitation in marine systems, we know little about the molecular mechanisms employed by co-limited microbes and how they may respond to a changing ocean environment^9^,^13^,^18^. For instance, increasing anthropogenic carbon dioxide (CO_2_) is decreasing seawater concentrations of both hydroxide and carbonate ions (OH^−^ and CO_3_^2−^), thereby reducing ocean pH (ref. 19). Hence, long-term ocean acidification is likely to have major consequences for key nutrient biogeochemical processes, including N_2_ fixation^20^. Past work has observed divergent responses of *Trichodesmium* spp. isolates to CO_2_ enrichment, suggesting that temporal CO_2_ fluctuations throughout Earth's history, perhaps combined with regional physicochemical forcings, could have resulted in differential ecotypic selection. This niche specialization relative to CO_2_ may have in turn influenced current relative abundances and biogeographic distributions of diazotrophic cyanobacteria^21^.

In a preceding long-term CO_2_ study, one cell line of IMS101 was split into one low (380 μatm) and one high (750 μatm) CO_2_ treatment and experimentally selected at each CO_2_ concentration for ∼4.5 years^22^. Intriguingly, constitutive increases in growth and N_2_ fixation rates in all high CO_2_-selected cell lines (750 selected) were observed following 4.5 years of selection, even after they were switched back to 380 μatm CO_2_ for 2 years. These findings help to reveal the potential responses of a key nitrogen biogeochemical cycle process to the evolutionary consequences of natural selection by future ocean acidification. However, virtually nothing is known about how adaptation of *Trichodesmium* to changing CO_2_ will in turn interact with the pervasive, long-term Fe and P co-limitation implied by *in situ* observations^1^.

To begin to address these issues, we examine the cellular responses of *Trichodesmium erythraeum* strain IMS101 (hereafter IMS101) to Fe and/or P (co)-limitation using a global proteomics approach in the context of long-term adaptation to both current CO_2_ concentrations and projected future ocean acidification conditions^23^. We subjected both the aforementioned 380- and 750-selected cell lines^22^ to long-term (∼1 year) Fe/P co-limitation selection, followed by either Fe or P additions to Fe/P co-limitation-selected subcultures, to generate steady-state Fe and P single-limitation treatments. Our results demonstrate a complex response of cellular metabolism specific to Fe/P co-limitation, which includes increased growth rates, broad proteome restructuring and cell size reductions relative to growth limited by a single nutrient. This global cellular response may have resulted from long-term selection by widespread Fe/P co-limitation, whereby cell size reductions help to relieve both diffusion and ligand-exchange kinetic limitation, thereby facilitating increased growth^24^. Reduced elemental quotas of smaller cells may also allow cells to maintain more rapid division rates when multiple resources are limiting^22^. Furthermore, elevated CO_2_ interacting with Fe/P co-limitation induces additional proteome shifts relative to the present day CO_2_, characterized by increased abundances of proteins involved in broad cellular metabolic functions. Together with increased growth rates, this restructuring reveals a unique co-limited phenotype under ‘balancing' co-limitation, in which simultaneous limitation by two nutrients may be more advantageous than ‘imbalanced' nutrient supply consisting of severe limitation by one resource and an excess of the other. This response fundamentally alters traditional interpretations of interactive nutrient limitations^10^ and their consequences for key global biogeochemical processes in both the present and future ocean.

## Results

### Growth and cell size

**Figure 1:**
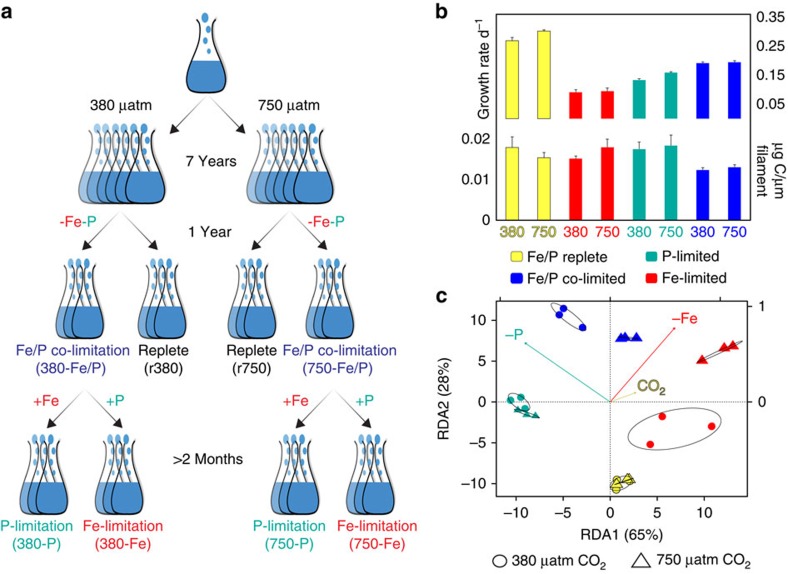
Experimental design with cell physiology and global proteome redundancy analysis. (**a**) Experimental design is displayed. (**b**) Cell-specific growth rates (top panel) and cell sizes (assessed using the proxy carbon content per filament length (μg C μm^−1^)) (bottom panel) are shown with error bars being s.e. (**c**) Redundancy (RDA) analysis of the global proteome. Colour key applies to both **b** and **c**. Ellipses are 95% confidence limits.

We generated Fe/P co-limited treatments from the 380- and 750-selected IMS101 cell lines, using semi-continuous culturing methods in biological triplicate for >1 year at each CO_2_ level (Methods). Following this extended co-limitation growth period, either Fe or P was added to subsamples of the Fe/P co-limited cell lines, which were then allowed to acclimate for ∼2 months before sampling, to create steady-state, triplicate Fe and P single-limitation treatments at both CO_2_ levels (Fig. 1a). This experimental design enables us to examine the effects of both short- and long-term nutrient limitation by Fe and/or P on protein biochemistry of *Trichodesmium* following ∼1,000 (380 selected) to ∼1,500 (750 selected) generations of selection by CO_2_. It has been observed that the mid-latitude oligotrophic oceans may be persistently Fe/P co-limited, a situation which is episodically relieved by pulses of either Fe or P from sources suh as atmospheric dust or advection^1^,^9^. Accordingly, our experimental design was intended to mimic these processes using diazotrophs adapted to both the present and future CO_2_ concentrations. Hence, we used these two CO_2_ concentrations to generate a hypothetical chronological progression examining interactions between Fe and/or P (co)-limitation scenarios and changing CO_2_ levels in *Trichodesmium*, from the present day up to the year 2100.

As previously observed, growth rates were significantly increased in high CO_2_ in both nutrient replete and P-limitation conditions^22^ (Fig. 1b). Conversely, no differences in growth rates were observed between the two CO_2_ regimes under either Fe limitation or Fe/P co-limitation (see Supplementary Note 2 for more discussion). This lack of elevated growth rates in high CO_2_/low Fe relative to low CO_2_/low Fe supports the results of prior studies with both a unicellular N_2_ fixer and *Trichodesmium*^25^,^26^ (see below and Supplementary Note 3 for more discussion).

Fe/P co-limited growth rates of IMS101 adapted to 380 and 750 μatm CO_2_ were lower than those of Fe- and P-replete cells, but were significantly increased relative to both Fe and P single-limitation treatments (by 100–110% and 22–43%, respectively, *P*<0.05) (Fig. 1b top). This growth response is consistent with previous results at current CO_2_ levels^18^. In addition, in the Fe/P co-limited cell lines, particulate organic carbon per unit of trichome length was 19–31% (380 selected) and 15–29% (750 selected) lower than in Fe-limited, P-limited and replete treatments, also similar to Garcia *et al.*^18^ (Fig. 1b bottom). When compared with either single-limitation treatment, this distinctive co-limitation phenotype suggests a large but unexplained advantage under a two nutrient ‘balancing limitation' regime, which is associated with reductions in cell size and volume. This demonstration of a substantial reproductive fitness advantage under Fe/P co-limiting conditions contrasts to the lower growth rate and biomass production typically seen under single nutrient limitations, and have been the focus of most prior work on diazotroph physiology.

### Interaction of CO_2_ and nutrient limitation on the proteome

Proteome analysis detected 1,908 proteins using a <1% false discovery rate (0.3% actual; minimum of 2 peptides per protein; see Methods), resulting in 307,509 spectra containing identified peptides from 24 discrete samples. Mapping to the ∼5,076 proteins predicted in the IMS101 genome^27^ yielded 37% coverage of the potential proteome (1,908/5,076). Relative protein abundances were measured by normalized spectral counts relative to total spectra collected across all 24 samples, with the normalization reflecting a very minor change.

To test for nutrient-specific proteome variation, nonmetric multidimensional scaling (Supplementary Fig. 1) and redundancy analysis (Fig. 1c) were applied to the normalized protein abundances, revealing consistent, nutrient-limited abundance patterns across replicate cultures for Fe/P replete, Fe-limited, P-limited, Fe/P co-limited, and/or high and low CO_2_ interactive profiles. Permutational multivariate analysis of variance was used to test for statistically significant correlations between limiting nutrient concentrations and proteome variation^28^, revealing limitation by Fe, P and Fe/P to all have individually significant effects on proteome variation (*P*<0.05). Elevated CO_2_ had no significant effect on the proteome in nutrient-replete treatments (*P*>0.05), despite inducing significant growth rate increases as previously observed^22^ (Fig. 1b and see Supplementary Note 2 for more discussion). However, the interactive effects of CO_2_ with each nutrient scenario (Fe, P or Fe/P) were all significant (*P*<0.05), suggesting that increased CO_2_ concentrations will significantly interact with nutrient-limited proteomes in the future ocean. Hence, the proteomes examined in this study strongly grouped by treatment and were significantly correlated to limiting nutrient concentrations, thus reflecting specific nutrient-limited or co-limited metabolisms. In particular, the distinctive segregation of Fe/P co-limited proteomes away from other single-limitation treatments (Fig. 1c and Supplementary Fig. 1) suggests a broad biochemical response underlying the concurrent increase in growth and decrease in cell size.

**Figure 2:**
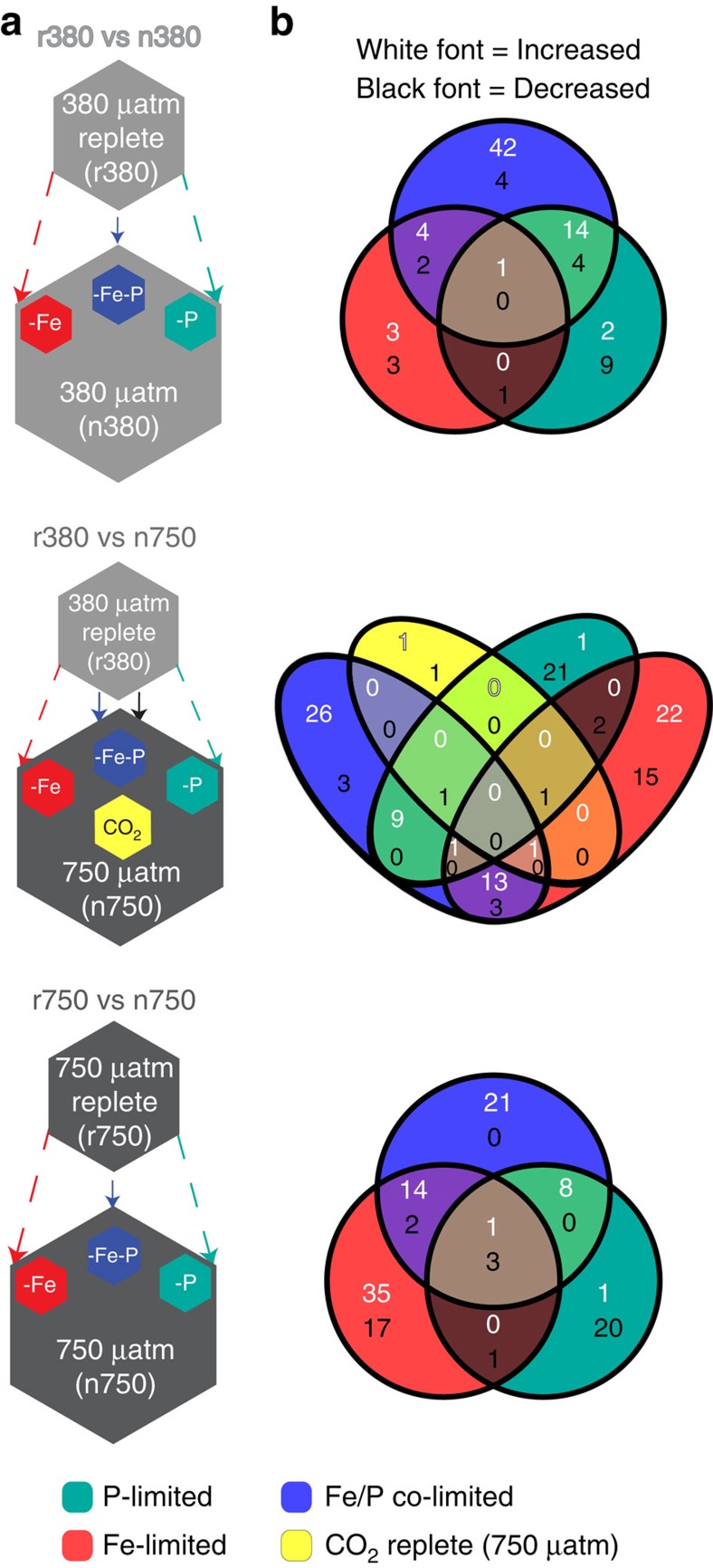
Experimental treatment comparative matrices and Venn diagrams of differential protein abundances. (**a**) Depiction of the method of differential abundance analysis (see Methods) with smaller hexagons (top) representing the replete reference treatments (r380 and r750) being compared with the nutrient limitation treatments (n380s and n750s, bottom). (**b**) Venn diagrams denote the relationships between the differentially abundant proteins from the analysis in **a** with proteins represented by the numbers therein. White font corresponds to proteins with significantly increased abundances relative to replete conditions and black font denotes significant decreases. The colour key applies to both **a** and **b**.

To identify proteins responding to specific nutrient/CO_2_ treatments, we tested for pairwise changes in protein abundances in each nutrient limitation scenario (P-limited, Fe limited and Fe/P co-limited) relative to either the replete 380 μatm CO_2_-selected (r380) or the replete 750 μatm CO_2_-selected (r750) controls using the Power Law Global Error Model^29^ with an estimated false positive rate of 10^−3^ (Fig. 2). In this hypothetical CO_2_ timeline, comparison of the r380 versus each nutrient-limited treatment in the 380 μatm CO_2_-selected cultures (n380s) reflects proteome changes due to nutrient (co)-limitation in the absence of elevated CO_2_ interactions, as in the present day scenario. Similarly, comparison of the r380 versus each nutrient treatment in the 750 μatm CO_2_-selected cell lines (n and r750s) illuminates how nutrient (co)-limited proteomes may interact with the shift to future higher CO_2_ conditions (*ca*. year 2100)^23^. Finally, analyses of the nutrient-replete and nutrient-limited, high CO_2_-selected cell lines (r750 versus the n750s) reflect proteome nutrient (co)-limitation responses between cell lines already adapted to high CO_2_ as in a future ocean. In addition, we compared Fe- and P-limited proteomes with either the 380 or 750 μatm Fe/P co-limitation (380-Fe/P or 750-Fe/P, respectively) treatments, to capture steady-state co-limited proteome changes in response to single nutrient inputs (Fe or P; see below). By assessing global proteome changes in this manner, we are able to track the effect of CO_2_ on the proteomes in each of the three nutrient limitation scenarios (Fe, P and Fe/P), while also elucidating the molecular mechanisms underlying cell size decreases and increased growth of the co-limited (Fe/P) phenotype relative to each of the two single-limitation treatments (P-limited or Fe-limited).

In the ‘r380 versus n380' scenario (Fig. 2a,b top), Fe/P co-limitation (380-Fe/P) retained the largest fraction of significantly increased protein abundances (∼93% of total increased protein abundances), whereas P limitation saw the largest amount of decreased abundances (∼61%). However, the interaction of elevated CO_2_ and nutrient limitation (for example, r380 versus n750s; Fig. 2a,b middle) drastically increased differentially abundant proteins in Fe limitation (750-Fe), suggesting increased CO_2_ to intensify Fe limitation as previously noted^22^ (also see below). In addition, numerous proteins (see below) that only increased in Fe/P co-limitation under 380 μatm CO_2_ (380-Fe/P) also increased in 750-Fe. Accordingly, in the ‘r750 versus n750' high-CO_2_ scenario (Fig. 2a,b bottom), Fe limitation dominated the increased protein fraction (∼63% of total increased protein abundances), whereas Fe and P limitation made up similar fractions of the decreased protein abundance pool (∼53% and 56%, respectively). Taken together, increasing CO_2_ in Fe limitation induces widespread changes in protein abundances, whereas in P limitation (750-P), decreases in protein abundances were primarily observed for this subset of the proteome.

### Proteins involved in nutrient transport and cell size

The ability to sustain Fe/P co-limited, increased growth relative to single limitation appears to be facilitated through general reductions in cell size, thereby alleviating high cell elemental quota requirements and physical nutrient acquisition limitations on large cells imposed by ligand-exchange kinetics and diffusion. As uptake rate per unit volume will vary inversely with cell diameter^24^, an increased surface area to volume quotient should help to relieve these limitations via increased transporter density per unit area, which can be assessed when transporter protein abundances are normalized to a proxy for cell size (μg C μm^−1^ filament length). Table 1 shows the average per cent changes in abundance for detected Fe and P stress proteins between the r380 and 380-Fe/P, the r380 and 750-Fe/P, and the r750 and 750-Fe/P before (circle) and after (asterisk) normalizing to μg C μm^−1^ filament length. Protein names in italics indicate protein abundances significantly affected by CO_2_, that is, from 380- to 750-Fe/P co-limitation conditions (Supplementary Fig. 2). Owing to cell size reductions seen under Fe/P co-limitation, transporter protein abundances increase per unit surface area relative to replete and single-limited cell sizes, which could explain the significant increases in co-limited but not single-limited growth rates. Hence, decreasing cell size under co-limitation enables cells to either increase or maintain uptake rates by either conserving or only marginally increasing particular transporter abundances (for example, IdiA and PstS) per unit area, thus facilitating energy and material reallocation away from protein synthesis and towards other processes.

**Figure 3:**
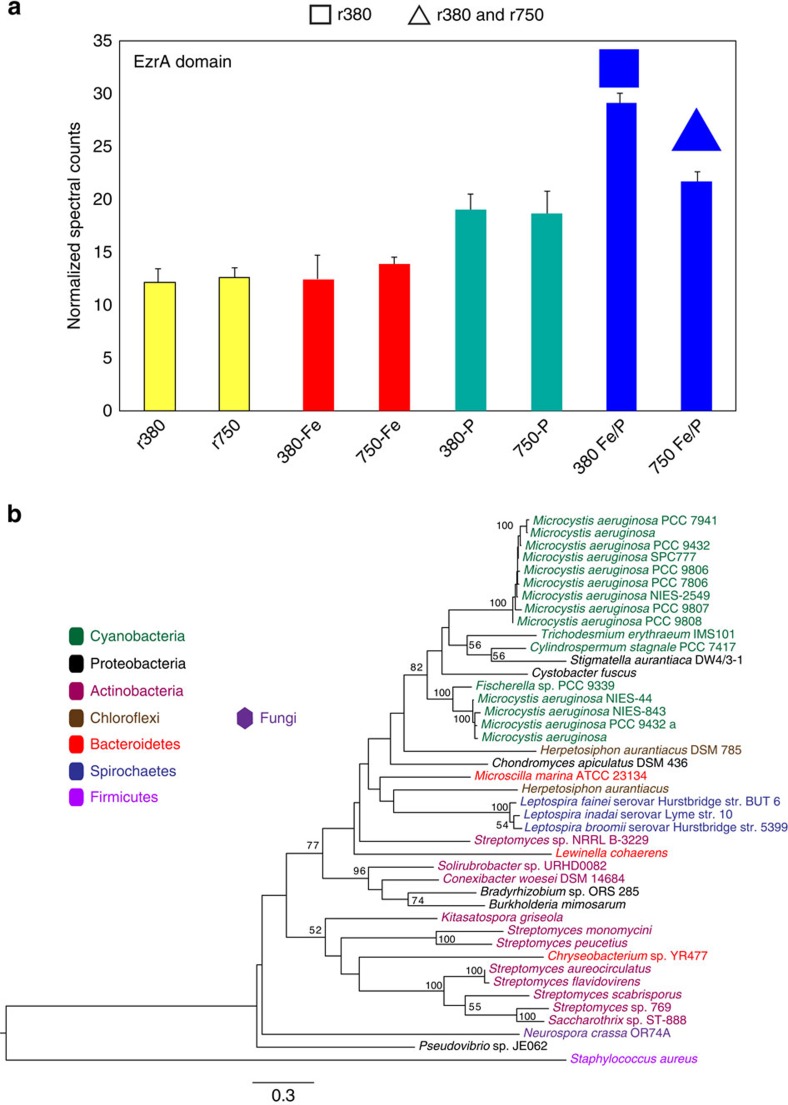
Normalized spectral counts of the EzrA-containing protein and its maximum likelihood phylogeny. (**a**) Shown are the EzrA-containing protein abundances across treatments. Colour key as in Fig. 1; squares indicate significance relative to the replete 380 and triangles relative to both the replete 750 and 380. Error bars are s.e. (**b**) Maximum likelihood phylogeny of detected protein homologues in NCBI (Methods) to the IMS101 EzrA-containing protein, with the *Staphylococcus* EzrA protein as the outgroup. Bootstrap values ≥50 are noted.

Biochemical evidence for cell size reduction specific to Fe/P co-limitation derives from a hypothetical protein (Tery_1090), which was found to include an EzrA (pfam06160) domain (web-based BLASTx)^30^. An EzrA-containing protein is required for regulating cell size in *Staphylococcus aureus*, as average cell diameter significantly increases after deletion of the EzrA protein^31^. This result is consistent with the lower cellular mass (Fig. 1b) and concurrent significant increase in Tery_1090 abundance exclusively under Fe/P co-limitation (Fig. 3a and Methods). Homologues (≥95% of the protein length with e-value <1e−10; see Methods) for Tery_1090 were detected in a handful of colony-forming bacteria based on a search of the current NCBI database (NCBI search using BLASTx; see Methods) and, of these, maximum likelihood phylogenetic analysis places Tery_1090 homologues among the small fraction of the colony-forming cyanobacterial diazotrophs (Fig. 3b). However, the EzrA domain itself is found in proteins distributed across a broad phylogenetic range of both unicellular and colony-forming bacteria whose global sequence homology to Tery_1090 (for example, *Staphylococcus* EzrA protein) fell well below our homologue threshold, suggesting adjacent sequences have considerably diverged over time, whereas the domain itself remained functionally conserved. The exact mechanism involved in the proliferation of the conserved EzrA domain remains to be determined but, nonetheless, the use of different and/or divergent cell size protein machinery with conserved domains suggests that there is strong selective pressure to maintain cell-size reduction capabilities in different habitats among a variety of distantly related bacteria. These data are consistent with prior observations of cell-size reductions with various nutrient-limiting treatments in other microbial systems^32^.

In addition, abundance profiles of other IMS101 orthologues to cell size/division proteins are generally consistent with prior observations in other bacterial systems^32^. For instance, cell size increases in *Escherichia coli* when the rod shape-determining gene, *mreB*, is inhibited^33^. Accordingly, an MreB protein homologue (Tery_1150) significantly increased in abundance in our IMS101 Fe/P co-limited cell lines, consistent with decreased cell size. Although the MreB protein and another cell division regulator, MinD, showed increased expression under Fe/P co-limitation, their expression also significantly increased under Fe single limitation, indicating strong control by Fe limitation despite there being no observed change in cell size under this single-limitation scenario (Supplementary Fig. 3 and Supplementary Data 1). Thus, although IMS101 does not significantly reduce cell size under Fe limitation alone, limiting Fe concentrations still impart some control over certain cell size/division machinery.

Sunda *et al.*^24^ observed decreases in both growth rates and cell size with decreasing Fe concentrations across a range of eukaryotic phytoplankton. In contrast, we saw reductions in growth, but not cell size, under Fe single limitation in *Trichodesmium*. Cell size only decreased once IMS101 was co-limited by both Fe and P, as in the unicellular diazotrophic cyanobacterium *Crocosphaera*^18^, whereas growth rate simultaneously increased relative to Fe-limited growth. Both this unicellular group and colony-forming N_2_-fixing cyanobacteria (*Trichodesmium*) share some cell size/division homologues, including MinD, which we observed was more abundant in IMS101 under both Fe/P co-limitation and Fe single limitation (above), despite the lack of cell-size changes under Fe limitation alone. Hence, either unknown mechanisms in diazotrophic cyanobacteria maintain cell size in the face of decreased growth under Fe limitation and/or nutrient-controlled mechanisms governing cell size reductions are only triggered under co-limiting conditions, leading to increased growth. It remains to be seen whether this coordination is specific to Fe/P co-limitation or whether other forms of co-limitation induce a similar response.

### Nutrient (co)-limited proteome profiles under increasing CO_2_

Both the single- (Fe or P) and co-limited (Fe/P) cell lines shared analogous abundance profiles of several well-characterized Fe or P nutrient stress proteins relative to the replete treatments, respectively (see below, and Supplementary Fig. 2 and Supplementary Data1). Intriguingly, once either Fe or P was added to co-limited cell cultures to achieve new single-limitation steady states, 71–86% and 97–100% of the differentially abundant Fe- and P-limited proteins exhibited significantly reduced abundances relative to their corresponding Fe/P co-limited abundances, respectively (Supplementary Data 2). These substantial fractions of reduced protein abundances following additions of either Fe or P to co-limited cells are accompanied by concurrent decreases in growth and increases in cell size in both Fe- and P-limited cells (Fig. 1b). Hence, the drastic decrease in protein abundances in cells that transitioned from Fe/P co-limited to single-limited steady states (Fe or P) may either be a product of reduced growth and/or a reallocation of energy towards cell-size increases at the cost of reduced growth. To tease apart the respective influences of P and Fe limitation on both the present day and future Fe/P co-limited protein biochemistry, we examined differences in protein composition between single and co-limited scenarios following selection by increasing CO_2_.

### P limitation versus Fe/P co-limitation under increasing CO_2_

P stress proteins (see below and Supplementary Fig. 2) were significantly more abundant in both the P-limited and Fe/P co-limited treatments relative to the replete cell lines, signalling P limitation under both conditions (see Methods). However, Fe/P co-limited cells significantly increased a large protein complement specific to co-limitation (Fig. 2b and see below). This difference between P limitation and Fe/P co-limitation steady states further highlights a broad, coordinated transition between single and co-limited states, with P limitation still persisting in both conditions.

There were also large differences between the 380-Fe/P and 750-Fe/P co-limited proteomes. For instance, adaptation to the interaction of elevated CO_2_ with Fe/P co-limitation (750-Fe/P) induced significant reductions in the abundance of particular P stress proteins, relative to 380-Fe/P (see Methods and Supplementary Fig. 2). Log_2_ fold changes ranged from −1.5 to −3.3 going from 380- to 750-Fe/P co-limitation for proteins involved in phosphonate acquisition (PhnD, PhnL, PhnK and PhnM), inorganic phosphate (P_i_) binding (SphX) and one protein of unknown function (Tery_3845) containing the P response regulator SphR motif, which is an orthologue to PhoB in *E. coli*^34^. In contrast, the high-affinity P_i_ uptake subunits of the Pst transporter complex, PstB (ATP binding) and PstS (P_i_ binding), remained unchanged in 750-Fe/P, as did the exopolyphosphatase enzyme, SurE (Supplementary Fig. 2). The *sphX* gene encoding the SphX subunit is an additional P_i_ binding subunit of the Pst transporter complex only found in a handful of cyanobacteria^34^ and is located upstream of the Pst transporter complex operon in IMS101, suggesting its regulation to be independent of the other Pst subunits. Specific reasons for both the reduction of the additional SphX subunit but not other Pst subunits, as well as other subunits of the phosphonate transporter complex in 750-Fe/P need further investigation. Nonetheless, this divergence in P-stress protein abundance suggests that increased CO_2_ may have varying effects on P stress complexes under Fe/P co-limitation, thus potentially affecting uptake efficacy of different forms of P.

### Fe limitation versus Fe/P co-limitation under increasing CO_2_

**Figure 4:**
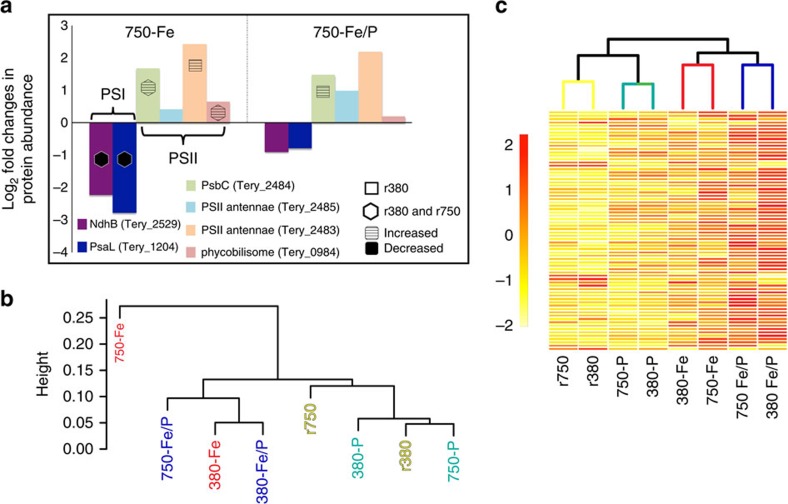
Log2-fold changes of photosystem proteins and hierarchical clustering of treatments using photosystem proteins or Fe/P complement proteins. (**a**) Shown is a bar graph of log2-fold changes of photosystem proteins in the 750-Fe and 750-Fe/P treatments, respectively. The symbols denote statistical significance relative to either the replete 380 (square) or both the replete 380 and 750 (hexagon). The striped fill denotes statistically significant increases in abundance relative to the replete condition(s) and significant decreases for the black fills. (**b**) Hierarchical clustering of treatments coloured by nutrient using Bray–Curtis dissimilarities calculated from photosystem protein abundances in **a**. (**c**) Hierarchical clustering of treatments with branches coloured by nutrient of Bray–Curtis dissimilarities calculated from protein abundances exclusively responding to Fe/P co-limitation (Fe/P protein complement). Heatmap scale bars represent individual protein abundances standardized across treatments.

Similar to published studies^35^,^36^, several Fe stress proteins (Fe starvation-induced protein A (IsiA and IsiB)) were enriched in both Fe-limited and Fe/P co-limited cell lines relative to the replete treatments (see Methods), thereby signalling general Fe limitation (Supplementary Fig. 2 and Supplementary Data1). The smaller amount of differentially abundant proteins observed in Fe single limitation under the present day CO_2_ (380-Fe) relative to a previous Fe-limitation study conducted at the present day CO_2_ (ref. 36) is likely to be a product of the different methods employed to generate Fe limitation, with Fe stress proteins still signalling Fe limitation in both studies. In particular, Fe single limitation in the present study was generated via P additions to cultures already acclimated to Fe and P co-limiting conditions for 1 year, which may impart fundamentally different physiological pressure relative to abruptly removing Fe from replete cultures as was done in Snow *et al.*^36^ and most other previous lab-based experiments^35^ (see Supplementary Note 3 for more discussion). Once increased CO_2_ interacted with Fe limitation (750-Fe), similar proteomic trends to Snow *et al.*^36^ involving major energy, carbon and nitrogen pathways were observed including a decreased photosystem (PSI:PSII) ratio (Fig. 4a and see below) and reduced fructose-1,6-bisphosphate aldolase abundance. In terms of nitrogen metabolism, AbrB (nitrogen uptake regulator), NifE (nitrogenase MoFe cofactor), NifH (nitrogenase) and glutamine synthetase all exhibited significant decreases (Supplementary Fig. 4 and see Supplementary Note 4 for more discussion). In addition, IsiA, IsiB and IdiA significantly increased in abundance in both 750-Fe and 750-Fe/P proteomes relative to the corresponding low CO_2_ treatments (see Methods and Supplementary Fig. 2). Taken together, these proteome shifts suggest that elevated CO_2_ intensified Fe limitation^22^ in *Trichodesmium* N_2_-fixing metabolism, irrespective of P concentration.

Fe limitation in high CO_2_-selected cell lines (750-Fe) caused significant decreases in abundance of all detected (PSI) proteins, as seen in previous present day CO_2_ Fe-limitation studies^36^,^37^, whereas all detected (PSII) proteins either increased or maintained abundance (Fig. 4a). However, under 750-Fe/P co-limitation, all PSI proteins exhibited abundances statistically indistinguishable from both the replete 380 and 750 treatments, thereby indicating PSI recovery under long-term co-limitation. In addition, 75% of detected, significantly increased PSII proteins in 750-Fe reduced their average abundances. Hence, the interaction of Fe limitation with high CO_2_-selected cell lines at steady state significantly reduces the PSI:PSII ratio, but this phenomenon is largely remedied under a Fe/P co-limiting regime. Hierarchical clustering of all detected photosystem components segregated Fe-limited treatments (Fe and Fe/P) away from both replete and P-limited conditions, implicating Fe limitation as a primary driver for the decreased PSI:PSII protein ratio even as P availability varies (Fig. 4b).

Hierarchical clustering of protein abundances exhibiting significant changes solely under co-limitation (the Fe/P protein complement; Supplementary Data 3) groups Fe single and Fe/P co-limitation treatments together, suggesting the Fe/P protein complement to be strongly influenced by Fe as well (Fig. 4c). In addition, numerous proteins that changed abundances exclusively under 380-Fe/P co-limitation also increased in abundance under 750-Fe (Fig. 2 and Supplementary Data 3). Accordingly, these trends in conjunction with the greater number of proteins shared between Fe single and Fe/P co-limitation (Fig. 2 and Supplementary Data 1 and 3) suggest the interaction of high CO_2_ adaptation and Fe limitation to be a primary driver of cell-size reduction and increased growth characterizing the Fe/P phenotype.

### Exclusive Fe/P co-limitation response

Of the differentially abundant proteins in 380-Fe/P co-limitation relative to the r380, 65% (*n*=46) were unresponsive in either Fe or P limitation alone (Fig. 2b top), which is evidence that this subset of proteins responds exclusively to co-limitation (Supplementary Data 3). Proteins with increased abundances constituted the majority of the Fe/P protein complement under all three comparative CO_2_ scenarios (86, 88 and 90%, respectively) with most showing no changes in abundance in single-limitation treatments (Fig. 2b). Although inherent growth rate-dependent differences between the nutrient limitations may contribute to these proteome shifts, the notably large amount of differentially abundant proteins (*n*=46) unique to 380-Fe/P co-limitation relative to the corresponding 380 single-limitation treatments (Fig. 2b, top) suggests the possibility of an evolutionarily conserved, coordinated biochemical response controlled by unknown regulatory systems underlying balanced limitation. This complex, distinct co-limitation response may have evolved due to intense selection by global Fe/P co-limitation regimes^1^,^14^.

Interestingly, although no growth rate differences were observed between the low and high CO_2_ treatments in Fe-limited (Fe and Fe/P) scenarios (Fig. 1b), large proteomic differences were observed between the 380-Fe and 750-Fe, as well as between the 380-Fe/P and 750-Fe/P (Figs 1c and 2b, Supplementary Fig. 1 and Supplementary Data 1 and 3). These differences suggest that these Fe-limited, CO_2_-induced proteome shifts are not growth rate driven, but instead are likely to be CO_2_ specific. In fact, they may represent the cellular compensatory mechanism(s) that allow *Trichodesmium* to maintain similar growth rates under Fe-limited conditions, regardless of changing CO_2_ levels. Taken together, these proteome shifts provide mechanistic insights into the departure of the co-limiting from the single limiting response as outlined in the Liebig model^10^. They also reveal changes to proteome architecture mediated by the interactions of CO_2_ and Fe limitation that are independent of growth rate.

COG (Cluster of Orthologous Genes) categories were assigned to the distinctive Fe/P co-limited protein complement (∼72% of the proteins; Supplementary Data 3 and see Methods). Although full pathway characterization was not possible, possibly due to potential limitations in protein detection, mapping these COG-assigned proteins to Kyoto Encyclopedia of Genes and Genomes pathways^38^ revealed them to reside in widespread cellular metabolisms (Supplementary Data 3). These included membrane stability and biogenesis, carbon catabolism and storage, cofactor biosynthesis, carbon fixation, photosynthesis and various precursor metabolisms, all of which together indicate a broad coordinated shift in numerous general cellular processes consistent with cell-size decreases and growth-rate increases.

**Figure 5:**
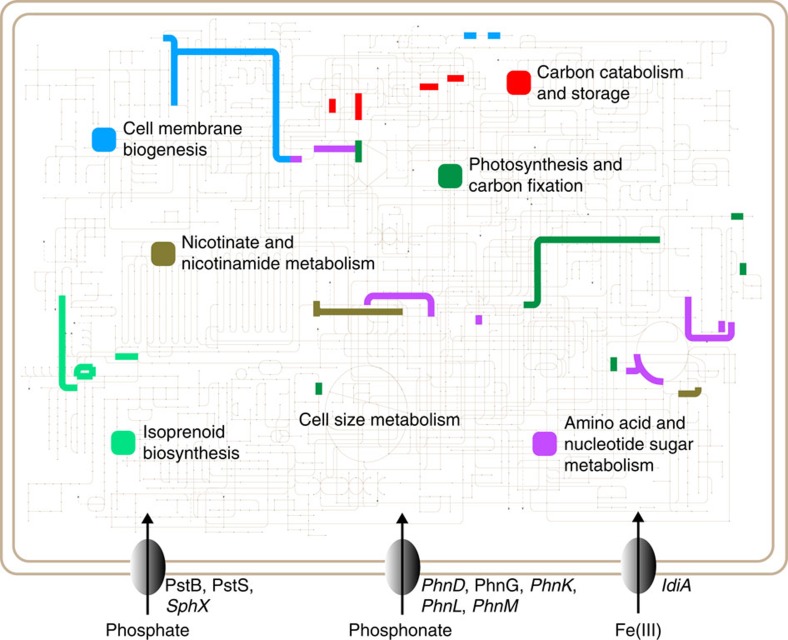
COG assigned proteins increased exclusively in Fe/P co-limitation mapped to Kyoto Encyclopedia of Genes and Genomes (KEGG) pathways. ‘Cell size metabolism' does not exist in KEGG pathways, but was added to acknowledge the cell size protein (Fig. 3a) exhibiting increased abundance in conjunction with these broad metabolic pathways under Fe/P co-limitation. Detected P and Fe transporter proteins are shown, indicating both Fe- and P-limitation, and italicized names are components affected by increased CO_2_ (see main text).

More specifically, the enrichment of proteins mapping to metabolisms involved in cofactor and precursor biosynthesis in concert with the increased abundances of both P- and Fe-limitation stress proteins suggests cellular reallocation under co-limitation to the biosynthesis of versatile precursor biomolecule residing at various metabolic junctures, possibly allowing for greater metabolic flexibility (Fig. 5). Cells experiencing (co)-limitation typically respond rapidly to nutrient additions, which involves global changes to cellular metabolism reflected in cell-size and growth-rate changes^39^. Hence, biosynthesis of general precursor molecules that can potentially be used by multiple pathways when nutrient fluxes are persistently variable may enable greater cellular plasticity and energy usage efficiency.

For example, increased protein abundance of isopentenyl pyrophosphate isomerase (IDI; Tery_1589) and squalene synthase (SQS; Tery_2043) suggests increased isoprenoid biosynthesis, which serve as critical components in various biochemical functions including quinones in electron transport chains, membrane components, photosynthetic pigments and others^40^. These isoprenoid enzyme increases are consistent with the concurrent increase in the protochlorophyllide reductase subunit ChlL (Tery_1532) and the protoporphyrinogen oxidase HemY (Tery_2218), where ChlL is involved in precursor production for chlorophyll (Chla) biosynthesis^41^ and HemY is involved in precursor production for both Chla and haems important for electron transport^42^. Furthermore, increased abundance of enzymes such as ornithine carbamoyltransferase (Tery_1323) involved in arginine/cyanophycin biosynthesis and hence nitrogen storage^43^ (Supplementary Fig. 3 and Supplementary Note 4), Glga (glycogen synthase; Tery_2147) involved in carbon storage, ManC and RfaE (lipopolysaccharide biosynthesis enzymes; Tery_1856, Tery_3495) involved in membrane stability and NadE (NAD synthetase; Tery_1984) involved in cofactors for photosynthesis and respiration, all corroborate the re-apportionment of cellular energy towards synthesizing flexible precursors and intermediates involved in a variety of pathways tied to core carbon metabolism (Supplementary Data 3).

Increased abundance of many precursor pathway proteins in Fe/P co-limited cells relative to Fe-limited and P-limited steady-state treatments could be related to the higher growth rates observed in the former condition (Fig. 1b). However, the replete treatments had significantly higher growth rates than Fe/P co-limited cultures (Fig. 1b), which were not reflected by higher levels of precursor pathway proteins, suggesting that this cannot be explained as a simple growth rate-driven phenomenon. Further investigations (for example, metabolite analyses) are necessary to validate the increased production of these intermediates under Fe/P co-limitation, but the increased abundance of these precursor biosynthesis proteins involved in various pathways looks to be a direct product of a metabolic shift under balancing Fe/P co-limitation not seen under single limitations.

The cellular regulation controlling these broad, coordinated proteomic shifts in widespread metabolic pathways under co-limitation is potentially controlled by upstream regulatory mechanisms at a whole systems level. For example, the switching of different RNA polymerase sigma factors (for example, ‘sigma switching') has been shown to aid in both stress and adaptive responses via transcriptional initiation of gene sets that are specific to particular environmental or internal cellular changes^44^. Predicted IMS101 sigma factors in the genome were either undetectable, not expressed or below our analysis threshold, thus preventing confident analysis of their differential abundances. However, future efforts can include more targeted studies looking at these proteins under nutrient limitation. In addition, other mechanisms such as DNA modifications^45^ (for example, epigenetics) and transposition^46^ have also been shown to aid in stress and adaptation, which in turn affect downstream transcription and translation. Hence, widespread changes to the proteome may be a product of coordinated changes from a smaller number of upstream regulatory systems, each controlling numerous biochemical pathways contingent on environmental stimuli. The results described here offer insight into downstream biochemical pathways affected by both independent and interactive nutrients and CO_2_, thus providing a foundation for future investigations of the regulatory mechanisms governing these biochemical changes.

The biochemical/physiological coordination described here offers the first molecular and mechanistic insight into the underlying cellular mechanisms governing the ‘balancing limitation' phenotype selected for by simultaneous Fe and P co-limitation. Looking ahead, the interaction of future increasing CO_2_ with single and multiple nutrient limitation scenarios induces fundamental metabolic shifts away from those seen under the present day CO_2_, including many responses that are clearly not simply growth rate driven. Surprisingly, numerous proteins (for example, photosynthetic proteins) that are differentially abundant under either Fe or P limitation are maintained at similar abundances to replete conditions under Fe/P co-limitation at high CO_2_. Concurrently, Fe/P co-limitation also induces a significant increase of a wholly different protein complement involved in core cellular functions including a variety of precursor metabolisms. The exact nature of this coordinated molecular and physiological response under Fe/P co-limitation needs further study, but the increased abundance of the Ezra-containing protein along with concurrent cell size reductions provides mechanistic insight into achievement of increased fitness (growth) relative to single-limitation scenarios.

Widespread co-limited oceanic conditions may have selected for a master regulatory pathway specifically evolved to sense co-limiting regimes, which may then affect downstream pathways to produce the co-limited phenotype. Alternatively, sovereign Fe and P regulatory mechanisms may initially respond independently to their respective limiting nutrients, followed by subsequent cellular coordination to produce the observed Fe/P co-limitation proteome profiles and phenotype. Regardless, it remains to be determined how widespread this response is among broad ranges of both prokaryotic and eukaryotic photoautotrophs. If other taxonomic groups exhibit similar co-limited responses, many classic nutrient limitation studies may need to be revisited, to determine what the ultimate consequences will be for controls on key global biogeochemical processes in the present day and future ocean.

## Methods

### Culturing methods

Stock cultures of *Trichodesmium* strain IMS101 were maintained at the University of Southern California, Los Angeles, California, USA, in modified Aquil medium without added combined nitrogen. They were grown at 26 °C under a light–dark cycle of 12:12 light:dark and maintained under a light intensity of 120 μmol photons per m^2^ s^−1^ incident irradiance. Experimental cultures were maintained in 0.2 μm-filtered, microwave-sterilized artificial seawater. Artificial seawater and Aquil nutrient stocks (except for the trace metal stock) were passed through a Chelex-100 column, to remove contaminating Fe before medium preparation.

Semi-continuous culturing methods using optically thin cultures were conducted to avoid nutrients becoming depleted before the next dilution^25^,^47^,^48^. All experimental conditions used three biological replicate bottles and each replicate was diluted individually according to growth rates calculated daily for that bottle using *in vivo* chlorophyll fluorescence measurements with a Turner 10 AU fluorometer^21^,^48^. For all experiments, final sampling occurred once steady-state growth (no significant difference in growth rates) was reached for at least ten generations and reported growth rates were calculated based on microscopic cell counts (see below).

To examine interactive effects of Fe and P limitation on growth of the cyanobacteria, *T. erythraeum* were grown in four treatments as follows: (1) Nutrient replete, 10 μM PO_4_^3−^ and 250 nM Fe; (2) P-limited, 0.25 μM PO_4_^3−^and 250 nM Fe; (3) Fe-limited, 10 nM Fe and 10 μM PO_4_^3−^; and Fe/P co-limited, 0.25 μM PO_4_^3−^and 10 nM Fe. EDTA concentrations were 25 μM irrespective of Fe conditions. Each of these four experimental nutrient treatments were generated using adapted cell lines that had been previously selected under 380 or 750 p.p.m. CO_2_ for ∼7 years^22^, for a total of 8 treatments. The replete and Fe/P co-limited cultures were grown in steady-state semi-continuous cultures for ∼12 months at each CO_2_ level. Following this long-term incubation, either Fe or P concentration was increased in subcultures of the Fe/P co-limited cell lines, thus creating cultures limited by either P or Fe alone, respectively. These two sets of single-nutrient-limited cultures were then grown at each pCO_2_ level for ∼2 months before being sampled together with the replete and Fe/P co-limited cultures.

Seawater medium was bubbled with 0.2-μm-filtered prepared air/CO_2_ mixtures (Praxair) to maintain stable targeted CO_2_ concentration treatments of 380 and 750 p.p.m. In-line high-efficiency particulate air filters were employed to avoid Fe contamination from particles in the gas tanks or lines and pH was monitored daily with dissolved inorganic carbon (DIC) being measured at the final sampling. Once steady-state growth was achieved, *Trichodesmum* filament abundance and lengths were measured in a 1-ml phytoplankton counting chamber using epifluorescence microscopy and significant differences between treatments were calculated using two-way analysis of variance along with Tukey's test. For proteome analysis, cultures were swiftly and gently filtered onto 5-μm polycarbonate filters (Whatman) during the middle of the photoperiod, immediately flash frozen and stored in liquid nitrogen until protein extraction.

### Carbonate buffer system analysis

DIC and pH were measured according to standard protocols^49^. DIC samples were collected from the experimental cultures and immediately poisoned with 200 μl of a saturated HgCl_2_ solution in 25 ml combusted borosilicate glass bottles. Samples were stored at room temperature until analysis on a UIC CO_2_ coulometer conducted in triplicate, as in Fu *et al.*^25^. Certified reference materials were used to calibrate total DIC measurements obtained from A. Dickson (UCSD). To confirm carbonate system equilibration, pH measurements were used for real-time monitoring and made using an Orion model 8102 combination electrode.

### Proteome analysis

Protein extraction, label-free mass spectrometry and spectral count normalization for global proteome analysis were performed as previously described^22^. The proteins and their normalized spectral counts can be found in Supplementary Data 4.

### Multivariate and pairwise analyses

Filtering of the proteome was performed before multivariate analysis, to eliminate consistently low normalized spectral counts across all treatments. In many cases where a protein contained zero counts in at least half of the treatments, the normalized spectral counts in the remaining treatments also proved to be uninformatively low (<3), which could skew ordination methods. However, certain proteins were only detected when a specific nutrient was limiting (for example, Tery_2498 and Tery_0463) in which case these proteins had zero spectral counts in half of the treatments as above but now consistently higher (>5) counts in treatments where that nutrient was limiting (for example, Fe). Thus, to remove proteins with little to no expression across all treatments, while simultaneously retaining proteins that showed substantial expression only in treatments where a particular nutrient was limiting, for each protein we summed the spectral counts across all replicates of all treatments (24 libraries) and divided by half (12) of the number of libraries. If this quotient was ≥5 normalized spectral counts, the protein was retained in the ordination analysis.

Nonmetric multidimensional scaling on normalized spectral counts of the filtered proteome was conducted in ‘R' (R Core Team 2014) using the ‘metaMDS' function from the vegan package^50^ with default settings (Bray–Curtis dissimilarities computed using Wisconsin double standardization) except for ‘autotransform=FALSE'. For the permutational multivariate analysis of variance, Bray–Curtis dissimilarities were calculated from the normalized spectral counts using the ‘vegdist' function and subsequently input into the ‘adonis' function in vegan with 2,000 permutations.

To analyse well-characterized nutrient stress proteins as indicators of cellular nutrient status, Welch's one-tailed *t*-tests assuming heteroscedasticity (unequal variances)^51^ were conducted in Microsoft Excel. Well-characterized nutrient stress proteins (Supplementary Fig. 2) are those supported by independent lines of empirical evidence^34^,^35^,^36^ representing a priori knowledge of their responses to either Fe or P limitation. Given this prior knowledge, statistically speaking these proteins represent ‘planned comparisons' defined as a selected subset of all possible comparisons that are not suggested by the current results but are predefined by prior theory in which an appropriate pairwise testing is a Welch's *t*-test^52^. In contrast, most other proteins lack a priori characterization; thus, their responses to environmental factors are unknown and require control of false-positive rates due to many independent exploratory tests, as in the PLGEM method (see below). *T*-tests were also conducted to test for significant differences between mean values of nutrient stress proteins that changed going from 380-Fe/P to 750-Fe/P.

Exploratory pairwise differential abundance tests were administered using a power law global error model provided in the PLGEM package with default settings^29^ and a delta (estimation of false-positive rate) of 0.001. Tests were conducted on normalized spectral counts between the nutrient-replete treatments and nutrient-limited treatments at each respective CO_2_. Following each pairwise test, each statistically significant protein within the differentially expressed pool was manually checked.

### Metabolic map

All proteins displaying differential expression exclusive to Fe/P co-limitation in each of the three CO_2_ scenarios (see main text) were matched to their corresponding COG IDs as per the IMG (Integrated Microbial Genomes) annotation (https://img.jgi.doe.gov). These COG IDs were used as input into the iPath2.0 tool^38^, to generate the metabolic map and beautified in Adobe Illustrator.

### Hierarchical clustering of protein expression

For all heatmaps, Bray–Curtis dissimilarities were calculated on normalized spectral counts using the ‘vegdist' function in the vegan package and hierarchical clustering was performed with the Heatplus package^53^ using the average (UPGMA) clustering method.

### Maximum likelihood phylogeny

The Tery_1090 protein containing the Ezra domain (see main text) was searched against the NCBI non-redundant database using the web-based BLASTx tool^30^ and hits with e-value <1e−10 and high scoring pairs covering >95% of the original query length were kept, to ensure robust homologue identification and multiple sequence alignments. The *S. aureus*^31^ Ezra protein was used as the outgroup. MAFFT was used for amino acid multiple sequence alignment^54^ with default settings and MEGA v6.06 (ref. 55) was used to construct maximum likelihood phylogeny with 100 bootstrap replicates and the following eight parameters: (1) substitution type=amino acid; (2) substitution model=wag with freqs. (+F) model; (3) rates among sites=gamma distributed (G); (4) no of discrete gamma categories=5; (5) gaps/missing data treatment=complete deletion; (6) ML heuristic method=nearest-neighbour interchange; (7) initial tree for ML=NJ/BioNJ; and (8) branch swap filter=very strong.

### Data availability

All protein spectral data used in the above analyses can be found in Supplementary Data 4.

## Additional information

**How to cite this article**: Walworth, N. G. *et al.* Mechanisms of increased *Trichodesmium* fitness under iron and phosphorus co-limitation in the present and future ocean. *Nat. Commun.* 7:12081 doi: 10.1038/ncomms12081 (2016).

## Supplementary Material

Supplementary Information Supplementary Figures 1-4, Supplementary Notes 1-4 and Supplementary References

Supplementary Data 1 Differentially abundant proteins in Fe-limitation relative to r380 and r750.

Supplementary Data 2 Differentially abundant proteins in Fe-limitation relative to 380-Fe/P or 750-Fe/P depending on the scenario

Supplementary Data 3 Proteins significantly changing in abundance exclusively in Fe/P co-limitation under either low or high CO2

Supplementary Data 4 Proteome Power Law Global Error Model dataset

## Figures and Tables

**Table 1:** Per cent changes in Fe and P stress proteins normalized to cell size.

	**r380 to 380-Fe/P**		**r380 to 750-Fe/P**		**r750 to 750-Fe/P**	
	•	★	•	★	•	★
*IdiA*	2	36	16	53	0.68	15
PstB	340	515	302	430	853	1,036
*PhnD*	388	603	137	223	121	158
*SphX*	193	306	84	142	104	138
PstS	21	70	25	68	29	50

Shown are the absolute average percent changes in Fe and P stress protein abundance going from the replete 380 to 380-Fe/P co-limited, the replete 380 to 750-Fe/P co-limited and replete 750 to 750-Fe/P co-limited before (circle) and after (asterisk) normalizing to cell size. Italicized protein names indicate significant changes in protein abundance under increased CO_2_ in Fe/P co-limitation.
