# Supplementary material for: Mechanisms of increased Trichodesmium fitness under iron and phosphorus co-limitation in the present and future ocean
Source: Nat Commun. 2016 Jun 27;7:12081. doi: 10.1038/ncomms12081 (PMC4931248; doi:10.1038/ncomms12081)
Supplement: Supplementary Information — Supplementary Figures 1-4, Supplementary Notes 1-4 and Supplementary References [file ncomms12081-s1.pdf]

Supplementary Figure 1

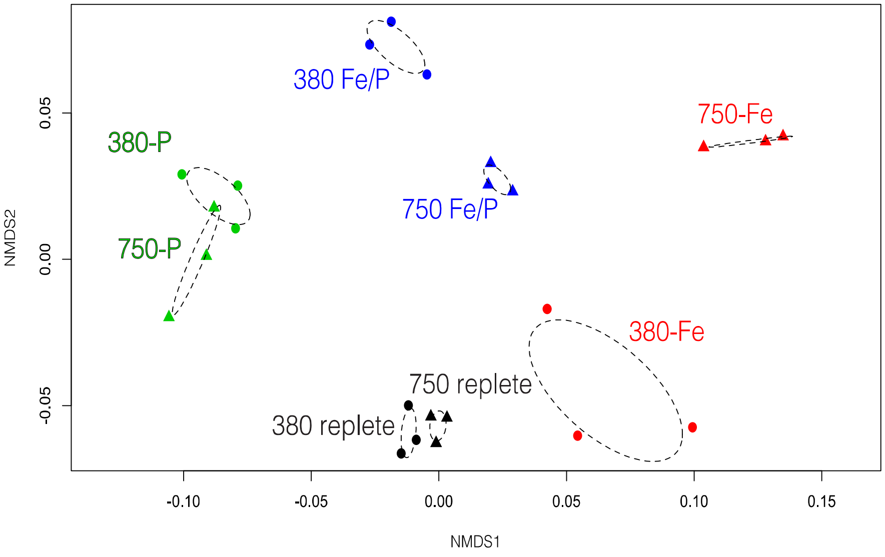

Supplementary Figure 1 **Nonmetric multidimensional scaling of the global proteome among all biological replicates of all treatments**. Ellipses are 95% confidence limits.

Supplementary Figure 2

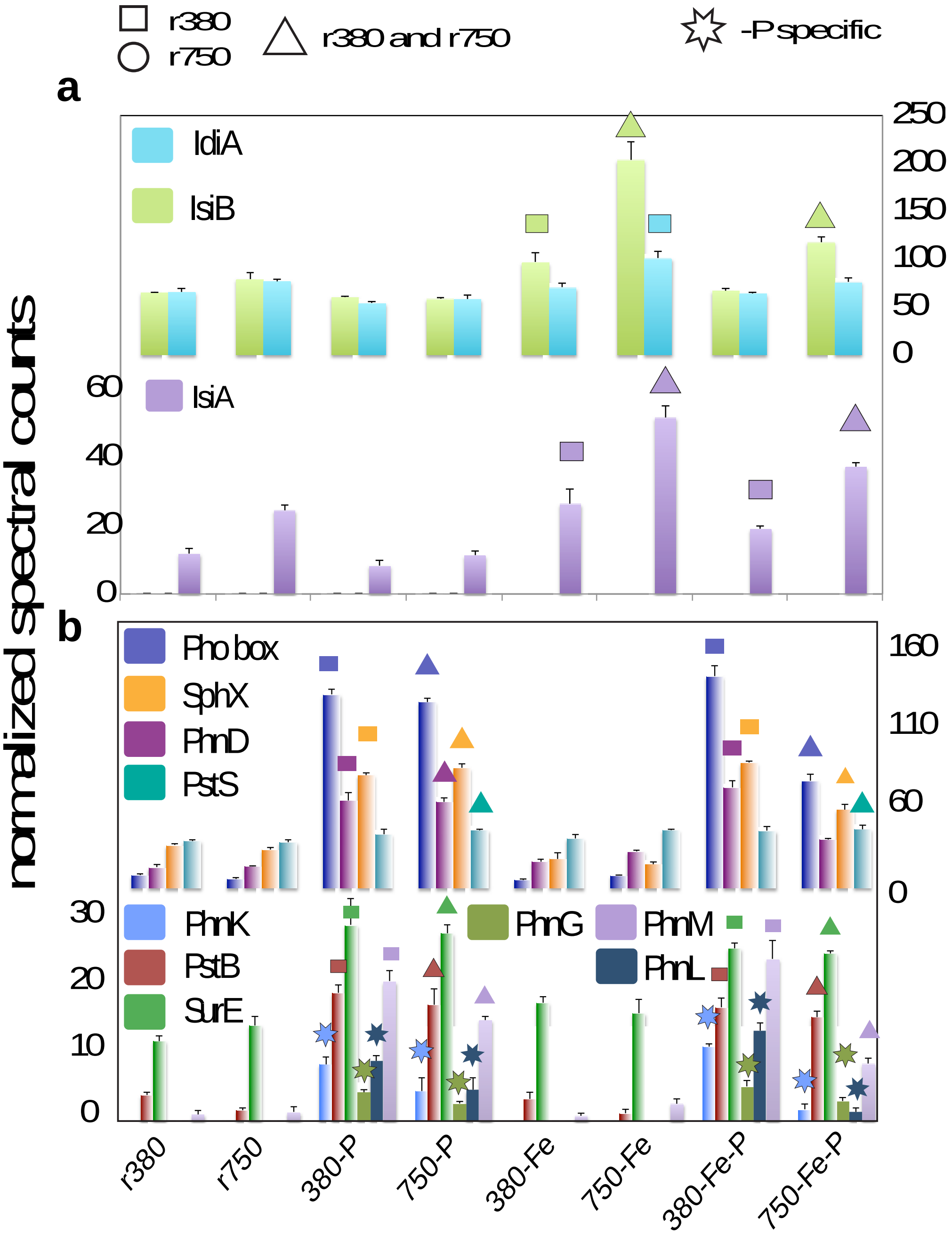

Supplementary Figure 2 **Protein abundances of Fe- and P-stress proteins in all conditions.** The symbols denote statistical significance relative to either the r380 (square) or both the r380 and r750 (triangle). The star denotes proteins only detected in phosphorus limitation. Error bars are standard errors.

Supplementary Figure 3

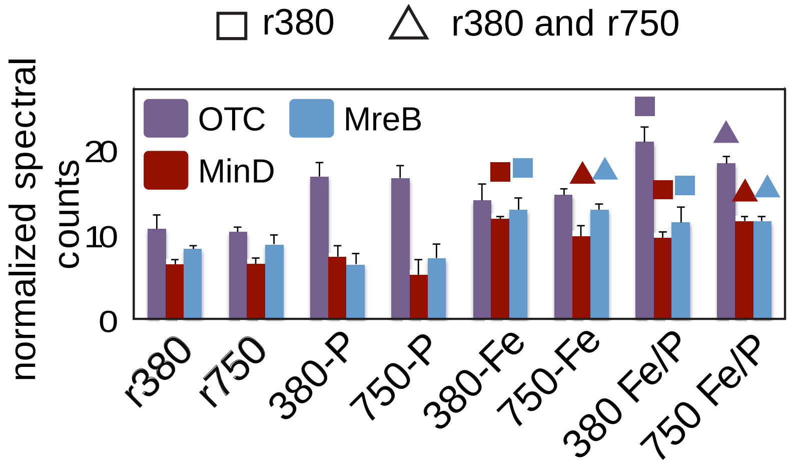

Supplementary Figure 3 **Protein abundances of select proteins**. The symbols denote statistical significance relative to either the r380 (square) or both the r380 and r750 (triangle). Error bars are standard errors. See main text and supplemental for discussion.

Supplementary Figure 4

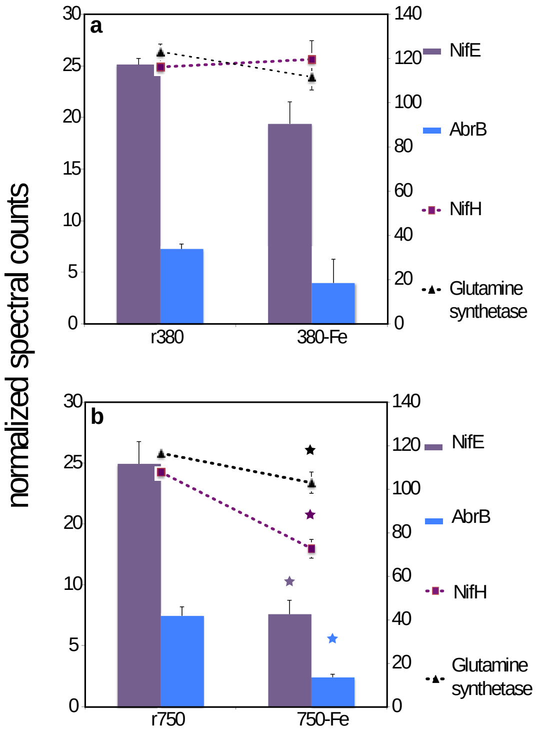

Supplementary Fig. 4 **Differentially abundant proteins involved in nitrogen metabolism.** Stars indicate statistical significance and error bars are standard errors. See main text and supplemental for discussion.

Supplementary Note 1

It is important to understand the interactive effects between ocean acidification and different types of nutrient limitation and how this interplay will influence diazotrophic physiology and ultimately, new N inputs and primary production. Dynamic forms of physiological nutrient limitation can be grouped into several categories and subcategories, which can be interrelated and concurrent. While Liebig's law of the minimum (Liebig limitation) states that a single resource limits overall yield, Blackman limitation refers to growth rate reduction rather than yield[1,2]. Since multiple inorganic nutrients are close to Liebig limitation in the oligotrophic oceans including nitrogen, phosphorus, and iron, both limitations can come into play simultaneously depending on respective concentrations and consumption rates. For example, carbon limitation of microbial photoautotrophs is traditionally considered as Blackman (rate) limitation since dissolved inorganic carbon (DIC) is quite high yet only ~1% of it exists as CO_2 while simultaneous low concentrations of either P or Fe can result in Liebig (yield) limitation due to their ephemeral inventories[2].

Defining the dynamic interplay between these two limitations can be obscuring especially in terms of co-limitation due in part to a strict interpretation of Liebig limitation where one nutrient is primarily limiting and the next most limiting nutrient becomes secondarily limiting rather than co-limiting[1]. However, true co-limitation can be more accurately defined as two or more limiting nutrients simultaneously influencing growth rather than sequentially. Hence, three different definitions of co-limitation have emerged that attempt to elucidate its biochemical underpinnings[2]. Type I aka independent nutrient co-limitation involves two potentially growth limiting nutrients that

are generally mutually exclusive in explicit biochemical function. Although most elements are generally interrelated on some level in the cell, this definition pertains to elements that are directly and functionally unrelated and has been used to describe, for example, nitrogen-phosphorus co-limitation. Type II or biochemical substitution co-limitation is defined as two elements that can substitute for the same biochemical role within the cell and can transpire in two different manners. The first happens when two elements can substitute effectively within the same enzyme as in zinc-cobalt co-limitation[3], and the second involves two enzymes that can carry out the same biochemical function but each utilizing a different element. Type III, or biochemically dependent co-limitation refers to an element becoming limiting as a result of the inability of the cell to sufficiently acquire the other element, as is the case with zinc-phosphorus co-limitation[4].

Supplementary Note 2

The minimal segregation of P-limited and nutrient-replete proteomes between CO_2 concentrations (Fig. 1c; Supplementary Fig. 1) indicates that the observed growth rate increases in high CO_2 under replete and P-limitation conditions may either be due to non-protein regulatory controls such as epigenetics[5], or to other portions of the undetected proteome.

Conversely, since both iron-limited treatments (Fe and Fe/P) had significantly differentiated proteomes based on CO_2 concentration (Fig. 1c, Supplementary Fig. 1), devoid of differences in growth rates between the two CO_2 adaptation regimes (Fig. 1b), the combination of high CO_2 with either Fe-limitation or Fe/P co-limitation seems to induce a broad biochemical response that is flexible enough to maintain growth rates irrespective of CO_2. This marked yet phenotypically undetected biochemical segregation

provides valuable insights into metabolic pathways that may come under biochemical pressure - and their corresponding cellular compensatory mechanisms - when iron is both limiting and co-limiting at future CO_2 levels.

Supplementary Note 3

Since the 380 and 750 µatm CO_2 cell lines were acclimated for ~7 years prior to the experiment, evidence of their adaptive departure from traditional short-term CO_2 physiologies as seen in other studies was shown through reciprocal transfers and diel N_2 fixation measurements[5]. Additionally, nutrient limited cell lines were allowed to acclimate for ~1 year to simultaneously limiting phosphorus and iron concentrations before being subjected to either Fe- or P-single limitation, respectively (See Methods). This long-term nutrient-limited steady state reflects a significant departure from traditional nutrient limitation experiments where typically, nutrients are either abruptly removed from replete cultures followed by brief acclimation or subsequently removed step-wise until cell death. Conversely, our measurements came after long-term exposure to simultaneous low iron and phosphorus concentrations, which may have altered proteome responses typically seen in short-term limitation studies as previously observed. For example, in a 3-week study where IMS101 was iron-limited in steady state under present day CO_2, nitrogenase (*nifH*) expression levels and enzyme activity began to recover towards the end of the experiment, which had not been previously observed in other shorter-term iron limitation studies under similar CO_2 regimes[6]. Similarly, adaptation to elevated CO_2 in the eukaryotic calcifying alga, *Emiliana huxleyi,* saw recovery of both growth rate and calcification paralleled by recovery of gene expression levels involved in pH regulation and carbon fixation[7]. Accordingly, other nutrient

responsive proteins may have undergone similar types of changes as a result of our 1-year acclimation.

Supplementary Note 4

Intracellular arginine concentrations have been shown to positively regulate net cyanophycin accumulation[8], which serves as a nitrogen reserve in diazotrophs[9]. Accordingly, the increased abundance of ornithine carbamoyltransferase (OTC, Tery_1323) involved in arginine biosynthesis may indicate increased cyanophycin and hence fixed nitrogen storage under Fe/P co-limitation (Supplementary Fig. 3). Increased N storage may in turn decrease the release of dissolved organic nitrogen (DON) into the water column[10] thereby possibly restricting new N input and exacerbating nitrogen limitation.

Average NifH expression maintained approximately similar expression levels in 380-Fe relative to the replete, but was significantly downregulated in 750-Fe. NifH protein levels and possibly other iron responsive proteins may have recovered in Fe-single limitation under ambient CO_2 concentrations due to the extensive acclimation period to low iron conditions as previously observed (Supplementary Note 3). Intriguingly, these enhanced protein changes in expression patterns were not reflected in differences in growth rates.

Supplementary References

1. de Baar, H. J. W. von Liebig's law of the minimum and plankton ecology (1899–1991). *Progress in Oceanography* **33,** 347–386 (1994).
2. Saito, M. A., Goepfert, T. J. & Ritt, J. T. Some thoughts on the concept of colimitation: Three definitions and the importance of bioavailability. *Limnol. Oceanogr.* **53,** 276–290 (2008).
3. Saito, M. A. & Goepfert, T. J. Zinc–cobalt colimitation of *Phaeocystis antarctica*. *Limnol. Oceanogr.* **53,** 1–11 (2008).
4. Arrigo, K. R. Marine microorganisms and global nutrient cycles. *Nature* **437,** 349–355 (2005).
5. Hutchins, D. A. *et al.* Irreversibly increased nitrogen fixation in *Trichodesmium* experimentally adapted to elevated carbon dioxide. *Nat. Commun.* **6,** (2015).
6. Küpper, H. *et al.* Iron limitation in the marine cyanobacterium *Trichodesmium* reveals new insights into regulation of photosynthesis and nitrogen fixation. *New Phytol.* **179,** 784–798 (2008).
7. Schluter, L. *et al.* Adaptation of a globally important coccolithophore to ocean warming and acidification. *Nat. Clim. Chang.* **4,** 1024–1030 (2014).
8. Stephan, D. P., Ruppel, H. G. & Pistorius, E. K. Interrelation between Cyanophycin Synthesis, L-Arginine Catabolism and Photosynthesis in the Cyanobacterium *Synechocystis sp.* strain PCC 6803. *Zeitschrift für Naturforschung C* **55,** (2000).
9. Li, H., Sherman, D. M., Bao, S. & Sherman, L. A. Pattern of cyanophycin accumulation in nitrogen-fixing and non-nitrogen-fixing cyanobacteria. *Arch. Microbiol.* **176,** 9–18 (2001).
10. Capone, D. G., Ferrier, M. D. & Carpenter, E. J. Amino Acid Cycling in Colonies of the Planktonic Marine Cyanobacterium *Trichodesmium thiebautii*. *Appl. Environ. Microbiol.* **60,** 3989–3995 (1994).
